# How inflammation dictates diabetic peripheral neuropathy: An enlightening review

**DOI:** 10.1111/cns.14477

**Published:** 2023-10-05

**Authors:** Yifan Cheng, Yinuo Chen, Kezheng Li, Shuwei Liu, Chunyang Pang, Lingfei Gao, Jiali Xie, L. V. Wenjing, Huan Yu, Binbin Deng

**Affiliations:** ^1^ Center for Rehabilitation Medicine, Department of Neurology, Zhejiang Provincial People's Hospital Affiliated People's Hospital, Hangzhou Medical College Hangzhou China; ^2^ Department of Neurology First Affiliated Hospital of Wenzhou Medical University Wenzhou Zhejiang Province China; ^3^ First School of Clinical Medicine Wenzhou Medical University Wenzhou Zhejiang Province China; ^4^ Department of Neurology, Shanghai East Hospital Tongji University Shanghai P.R. China; ^5^ Department of Geriatrics The Affiliated Hospital of Qingdao University Qingdao Shandong Province China; ^6^ Department of Pediatrics Second Affiliated Hospital and Yuying Children's Hospital of Wenzhou Medical University Wenzhou China

**Keywords:** diabetic neuropathy, inflammatory response, metabolic changes, molecular mechanism

## Abstract

**Background:**

Diabetic peripheral neuropathy (DPN) constitutes a debilitating complication associated with diabetes. Although, the past decade has seen rapid developments in understanding the complex etiology of DPN, there are no approved therapies that can halt the development of DPN, or target the damaged nerve. Therefore, clarifying the pathogenesis of DPN and finding effective treatment are the crucial issues for the clinical management of DPN.

**Aims:**

This review is aiming to summary the current knowledge on the pathogenesis of DPN, especially the mechanism and application of inflammatory response.

**Methods:**

We systematically summarized the latest studies on the pathogenesis and therapeutic strategies of diabetic neuropathy in PubMed.

**Results:**

In this seminal review, the underappreciated role of immune activation in the progression of DPN is scrutinized. Novel insights into the inflammatory regulatory mechanisms of DPN have been unearthed, illuminating potential therapeutic strategies of notable clinical significance. Additionally, a nuanced examination of DPN's complex etiology, including aberrations in glycemic control and insulin signaling pathways, is presented. Crucially, an emphasis has been placed on translating these novel understandings into tangible clinical interventions to ameliorate patient outcomes.

**Conclusions:**

This review is distinguished by synthesizing cutting‐edge mechanisms linking inflammation to DPN and identifying innovative, inflammation‐targeted therapeutic approaches.

## Graphical abstract

Understanding the role of inflammation in diabetic peripheral neuropathy's progression.

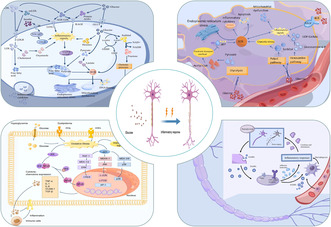

## 1 INTRODUCTION

Diabetic neuropathy is one of the significant complications of diabetes and occurs in approximately 50% of diabetes patients.^1^, ^2^ Diabetes is a global epidemic, its prevalence continues to increase, and it is expected to affect 693 million adults by 2045,^3^, ^4^ which means a corresponding increase in the incidence of diabetic neuropathy. Diabetic neuropathy affects different parts of the nervous system and presents multiple manifestations. The common forms of diabetic neuropathy are distal symmetric polyneuropathy (DSPN) and autonomic neuropathy.^5^ The past decade has seen rapid developments in understanding the complex etiology of diabetic neuropathy.

The pathogenesis of diabetic neuropathy is very complex. Hyperglycemia, dyslipidemia, and insulin resistance trigger a cascade of responses, activating pathways such as the polyol pathway, glycolysis pathway, hexosamine pathway, and advanced glycation end‐product pathway. These activations enhanced oxidative stress and inflammatory signals, leading to endoplasmic reticulum stress, mitochondrial dysfunction, DNA damage, and elevated inflammatory factor levels. Ultimately, this complex process substantiates the onset of diabetic neuropathy.^2^, ^5^, ^6^, ^7^ Despite the confirmation of these mechanisms through clinical and experimental studies, the quest for effective treatments remains ongoing. Therefore, clarifying the pathogenesis of diabetic neuropathy and finding effective therapies are crucial issues for diabetic neuropathy.

In this review, we summarize the current knowledge on the pathogenesis of diabetic neuropathy, especially how metabolic factors affect the course of the disease and the indispensable role of the inflammatory response. Furthermore, we touch on recent advances in therapeutic strategies.

## 2 PATHOGENESIS OF DIABETIC NEUROPATHY

**FIGURE 1:**
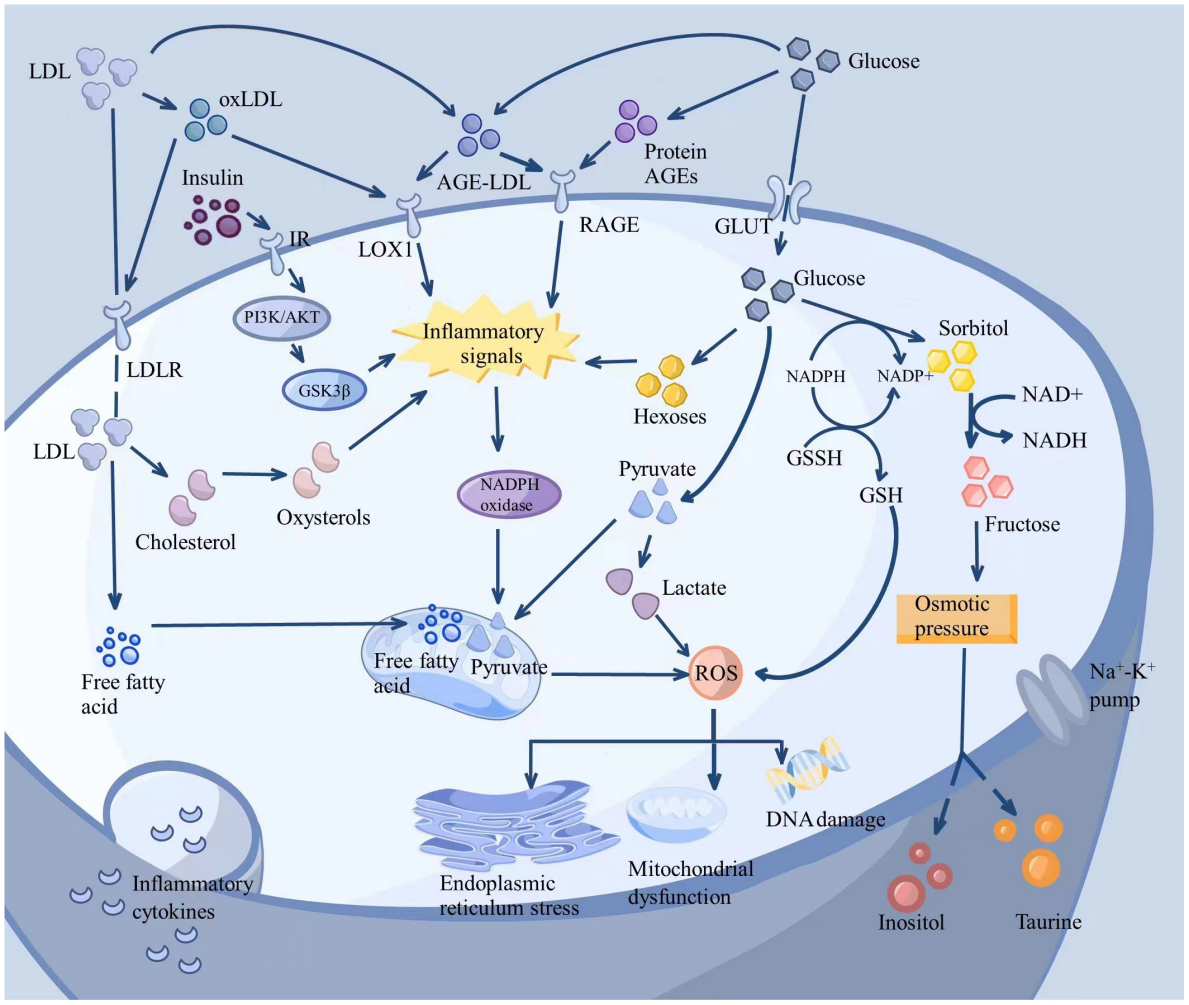
The pathogenesis of diabetic neuropathy. Hyperglycemia, dyslipidemia, and insulin resistance cause oxidative stress and inflammatory response, induce pathological alterations including DNA damage, endoplasmic reticulum stress, and mitochondrial dysfunction, and ultimately lead to diabetic neuropathy.

The pathogenesis of diabetes involves insulin resistance in tissues (such as adipose tissue, muscle, and liver) and insufficient insulin secretion in pancreatic cells,^8^, ^9^ which may lead to hyperglycemia. Thus, hyperglycemia is associated with the development of diabetic neuropathy. However, the cause of diabetic neuropathy is very complex and involves hyperglycemia, dyslipidemia, and insulin resistance (Figure 1).

**FIGURE 2:**
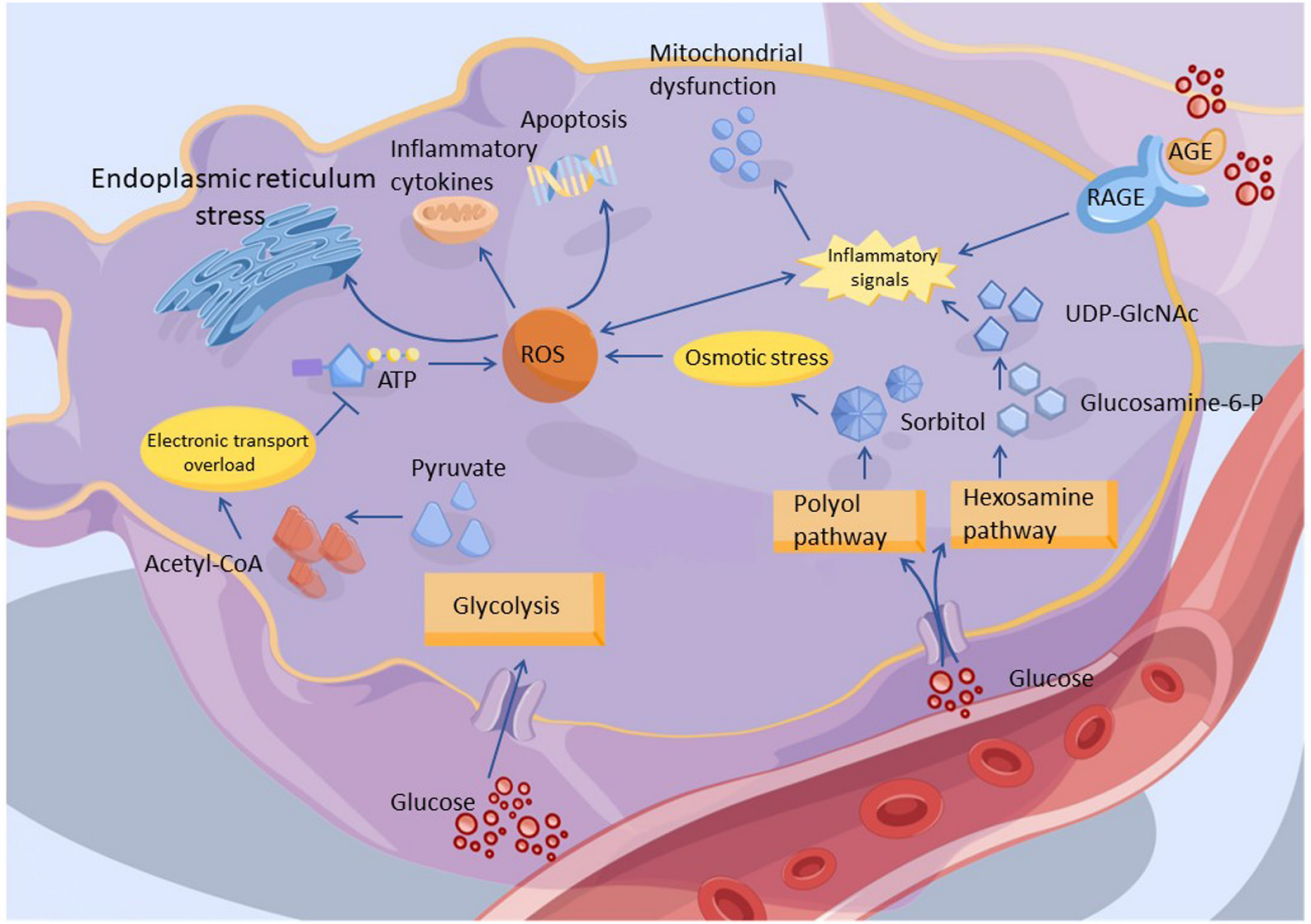
Hyperglycemia initiated injury mechanisms in neurons. Intracellular glucose is metabolized through the process of glycolysis principally. Besides, excess glucose activates advanced glycation end products (AGEs) formation and the polyol and hexosamine pathways. They cause an imbalance in the mitochondrial redox state, lead to excess formation of reactive oxygen species (ROS), and promote inflammatory response that result in neuronal injury.

High blood sugar, or hyperglycemia, is a crucial contributor to diabetic neuropathy, primarily through its damaging effects on nerves. This damage is propagated via multiple biological pathways (Figure 2).^3^, ^10^, ^11^, ^12^ First, hyperglycemia excessively activates the glycolytic pathway in nerve and Schwann cells, leading to an accumulation of lactate and pyruvate.^13^, ^14^ This accumulation causes oxidative stress and mitochondrial damage due to an increased voltage gradient on the mitochondrial membrane.^15^, ^16^ Second, the polyol pathway, triggered by high blood sugar, results in an excessive buildup of sorbitol and fructose, activating the intracellular osmotic stress pathway. This pathway disrupts neuron function and its response to oxidative stress through an imbalance in myoinositol efflux and excessive consumption of NADPH.^17^, ^18^, ^19^ Third, excessive glucose can activate the hexosamine pathway, resulting in gene expression changes through the conversion of fructose‐6‐phosphate into uridine diphosphate N‐acetylglucosamine (UDPGlcNAc) and its subsequent binding to transcription factors.^20^, ^21^, ^22^ Finally, chronic hyperglycemia facilitates the formation of advanced glycation end products (AGEs) which alter protein structure and function. The binding of AGEs to their receptors in neurons and Schwann cells triggers an inflammatory response and additional oxidative stress.^23^, ^24^, ^25^

Studies have shown that dyslipidemia accelerates the progression of neuropathy, even in patients in the early stages of diabetes.^26^, ^27^ Dyslipidemia, especially triglycerides, and cholesterol are considered neurotoxic. Neuronal damage caused by dyslipidemia is associated with free fatty acids (FFA) and oxidized low‐density lipoprotein (ox‐LDL).^27^, ^28^ Ox‐LDL and glycated LDL bind to neuronal receptors, typically LDL receptor 1 (LOX1) and Toll‐like receptor 4, triggering inflammatory signals and increasing oxidative stress responses.^29^, ^30^ High levels of FFA lead to insulin resistance and hyperglycemia and enhance the complications of diabetes. FFA also induces mitochondrial dysfunction and accumulation of reactive oxygen species through β‐oxidation and lipid toxicity.^31^ Studies also show that high‐density lipoprotein cholesterol is associated with the later development of diabetic neuropathy.^32^ It is also the only negative predictor of risk for diabetic foot ulcers.^33^ In addition, higher levels of sphingolipids DSLs, associated with diabetic neuropathy, were found in patients with type 2 diabetes mellitus (T2DM), highlighting the intricate connection between lipid metabolism and diabetic neuropathy.^34^

Insulin resistance (IR) plays a crucial role in the pathogenesis of diabetic neuropathy, and IR has a more significant role in T2DM neuropathy.^35^ Low vitamin D levels appear to be associated with insulin‐resistant diseases, such as T1DM, T2DM, and diabetic neuropathy.^36^ IR cross‐influences inflammatory responses, oxidative stress, and ectopic lipid accumulation in the liver and skeletal muscles.^37^, ^38^, ^39^ At the cellular level, insulin resistance affects diabetic neuropathy by damaging insulin signaling in postganglionic neurons and disrupting lipid metabolism in Schwann cells through the PI3K / AKT / mTOR pathway.^40^

## 3 ROLE OF THE INFLAMMATORY RESPONSE IN DIABETIC NEUROPATHY

**FIGURE 3:**
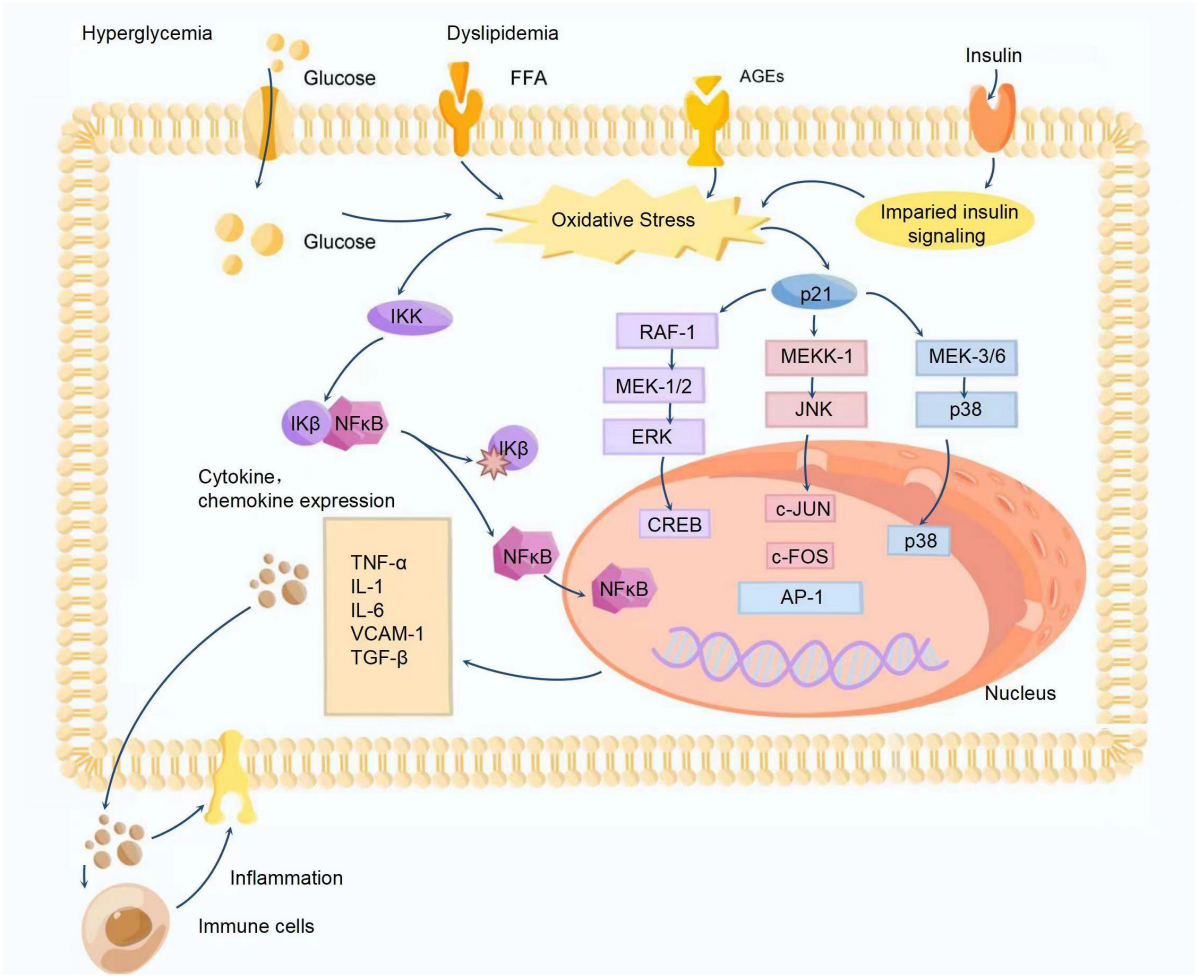
The role of DAMPs in inflammation‐mediated diabetic neuropathy. Chronic exposure to hyperglycemia damages neurons, resulting in the release of intracellular damage‐associated molecular patterns (DAMPs) into the extracellular space. DAMPs are recognized by PRRs such as Toll‐like receptors. Activation of these receptors leads to inflammatory responses and progressive nerve injury.

Inflammation is an adaptive response triggered by noxious stimuli and conditions.^41^ Tissue stress or malfunction may induce mild chronic inflammatory activity by activating the innate immune system.^42^ Evidence shows that long‐term mild inflammation has a significant negative impact on the pathogenesis of diabetic neuropathy (Figure 3).^43^, ^44^ Generally, glucose, lipoproteins, and oxidized and glycated proteins bind with various receptors on neurons. These receptors include transporters that internalize glucose and lipids, which increase the accumulation of glucose and lipids in cells and disrupt mitochondrial metabolic pathways. These receptors also initiate inflammatory signals.^6^, ^18^, ^45^ Immune cells, cytokines, chemokines, soluble adhesion molecules, and inflammation‐related biomarkers are increased in peripheral nerve or cerebrospinal fluid samples from patients with diabetic neuropathy.^46^, ^47^, ^48^, ^49^, ^50^, ^51^, ^52^ Understanding the role of key inflammatory pathways and molecules in diabetic neuropathy will help discover anti‐inflammatory approaches that could potentially inhibit the development of neuropathy. This section summarizes the inflammatory cells, molecules (including inflammatory cytokines, chemokines, and adhesion molecules), and critical inflammatory pathways (the NF‐κB pathway and the MAPK pathway) involved in developing diabetic neuropathy.

### 3.1 Immune cells in DPN

In T1DM individuals with pain compared to those without, a proportional increase in CD4+ central memory T cells and an absolute increase in classical and nonclassical monocytes is observed among peripheral blood immunophenotypes.^53^ Calcitonin gene‐related peptide (CGRP)‐specific CD4 T cells may be a potential marker for TIDM. Given the widespread presence of CGRP in the nervous system and its critical role in developing peripheral neuropathic pain, CGRP is likely to play an important role in diabetic neuropathy.^54^ In the early and late stages of disease in STZ‐induced T1DM model animals, significant neutrophil infiltration was found in the dorsal root ganglia (DRG). T‐cell infiltration in the DRG was prominent in the late phase.^55^ Sciatic nerve samples from T2DM patients showed macrophage and T‐cell infiltration and increased expression of clusters of cells expressing CD40, a critical, crucial molecule in the upregulation of HIF‐1α and PTEN underlying microangiopathy in diabetic nerve pathology.^56^

**FIGURE 4:**
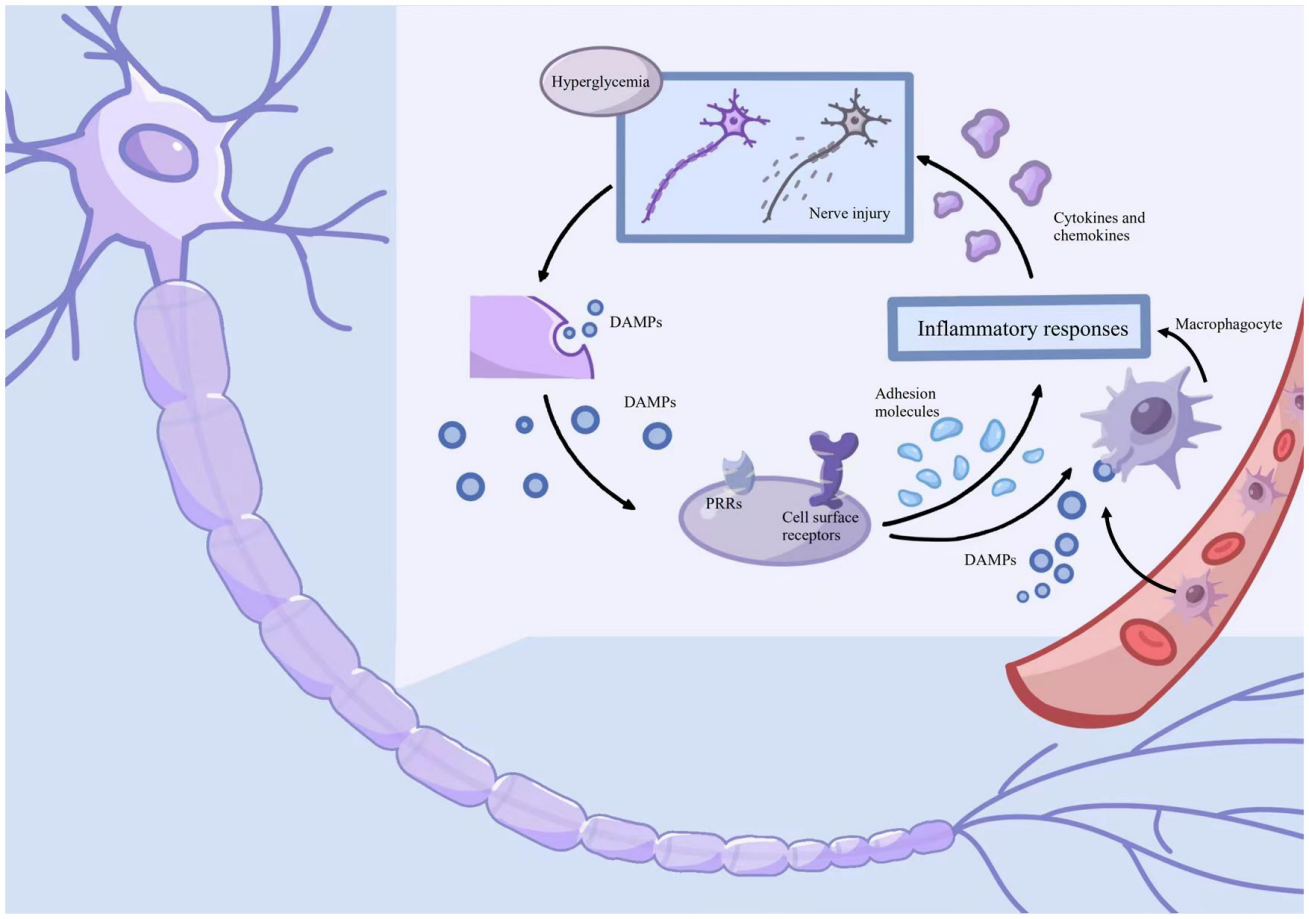
Inflammatory response implicated in the pathophysiology of diabetic neuropathy. Hyperglycemia and dyslipidemia together with insulin resistance result in the activation of stress and inflammatory pathways (NF‐κB and MAPK) leading to widespread changes in gene expression and upregulation of cytokines/chemokines, and soluble adhesion molecules, which further leads to the recruitment of immune cells, augmenting the inflammatory response.

The innate immune system utilizes distinct classes of pattern recognition receptors (PRRs), which recognize danger‐associated molecular patterns (DAMPs) to initiate downstream inflammatory cascades (Figure 4).^57^ Toll‐like receptors (TLRs) are PRRs.^58^ Emerging evidence shows TLR‐mediated inflammatory cascades contribute to diabetic neuropathy.^59^ TLR4 is highly expressed in endoneurial vessels of sciatic nerves in leptin‐deficient ob/ob mice.^60^ TLR4 downregulation ameliorated hyperalgesia and mechanical allodynia in an experimental diabetic neuropathy model.^61^, ^62^ TLR2 is an important regulator that promotes the polarization of inflammatory macrophages, and TLR2 inhibitors were found to suppress inflammation and promote M2 phenotype macrophages in sciatic nerves from T2DM mice.^63^

Among innate immune cells, macrophages have been primarily investigated in diabetic neuropathy. A growing body of evidence has shown that macrophage polarization, according to their microenvironment, plays an essential role in the development of diabetes. M1 phenotype macrophages that promote inflammation favor glycolysis, produce lactate instead of metabolizing pyruvate to acetyl‐CoA, and have a tricarboxylic acid cycle interrupted at two points. M2 phenotype macrophages that downregulate inflammation use the beta‐oxidation of fatty acids and oxidative phosphorylation to create energy‐rich molecules.^64^ The recruitment of blood monocyte‐derived macrophages in the spinal cord was found to promote the development of painful diabetic neuropathy in diabetes.^65^

### 3.2 Role of inflammatory cytokines in diabetic neuropathy

Cytokines are low‐molecular‐weight glycoproteins that are secreted mainly, but not exclusively, by immunological cells such as T cells, macrophages, and neutrophils. Other cells that secrete cytokines include Schwann cells and glial cells of the central nervous system. Proinflammatory cytokines, such as TNF‐α, IL‐1, IL‐6, IL‐8, monocyte chemoattractant protein‐1, and C‐reactive protein, are mainly produced by activated immune cells. Proinflammatory cytokines were reported to play a critical essential role in developing diabetic neuropathy. Table 1 lists clinical studies assessing the relationship between markers of inflammation and diabetic neuropathy (Table 1).^44^, ^50^, ^51^, ^66^, ^67^, ^68^

**TABLE 1:** Studies assessing the relationship between markers of inflammation and diabetic neuropathy.

Study	Protocol	Biomarkers	Main finding	Ref.
González Clemente 2005	120 patients with T1DM in Spain, 36 had diabetic neuropathy and 84 did not.	sTNFR1, sTNFR2	Higher sTNFR1 and sTNFR2 in diabetic neuropathy compared to T1DM without diabetic neuropathy.	^50^
Doupis 2009	212 individuals aged 21–80 years, 55 healthy control subjects, 77 diabetes patients with DPN, and 80 diabetes patients without DPN. The neuropathic group was subsequently subdivided into patients with and without neuropathic pain.	CRP, TNFα, fibrinogen, RANKL, OPG, IP‐10/CXCL10, RANTES/CCL5, and leptin	Higher CRP, TNFα, fibrinogen, OPG, and leptin in diabetes patients with DPN compared to diabetes patients without DPN. Higher sICAM‐1 and CRP in patients with painful neuropathy compared to painless neuropathy.	^51^
Zheng 2021	315 T2DM patients in China, with 6.5 years follow‐up, evaluate the predictive effect of cytokines on incidence of DPN.	IL‐6, TNF‐α, VEGF, ICAM‐1	Increased TNF‐α and ICAM‐1, predicted the incidence of DPN over 5 years in Chinese diabetes patients.	^88^
KORA F4 Study 2013	Determine serum concentrations of mediators of subclinical inflammation among 1047 participants 61–82 years of age in the German KORA F4 survey.	CRP, IL‐6, IL‐18, TNFα, IL‐1RA, adiponectin	Higher IL‐1RA in DPN patients; positive association of IL‐6 and IL‐1RA with MNSI score.	^89^
KORA F4 Study 2015	measured circulating levels of seven immune mediators and assessed their associations with the presence of painful DSPN in individuals with painless (*n* = 337) and painful DPN (*n* = 54) from participants aged 61–82 years (n = 1047) in the German KORA F4 survey.	CRP, IL‐6, IL‐18, TNFα, IL‐1RA, adiponectin	Higher IL‐6 in painful compared to painless DPN.	^90^
KORA F4/FF4 Study 2017	530 participants in Germany aged 62–81 years with 6.5 years follow‐up; evaluate the predictive effect of cytokines on incidence of DPN and DPN progression.	CRP, TNFα, IL‐6, IL‐1RA, IL‐18, sICAM‐1, adiponectin, omentin	Higher IL‐6 and TNF‐α associated with incident DSPN; sICAM‐1 and IL‐1RA were positively associated with progression of DPN	^70^
The German Diabetes Study	Motor and sensory NCV was assessed in 352 individuals with T2DM and 161 individuals with T1DM	CRP, IL‐6, IL‐18, total adiponectin, HMW adiponectin, HMW/total adiponectin	High‐serum IL‐6, HMW adiponectin, total adiponectin, and their ratio were associated with the presence of DPN and reduced motor NCV in T2DM. In T1DM, only HMW and total adiponectin showed positive associations with motor NCV.	^91^

#### 3.2.1 Tumor necrosis factor‐α (TNF‐α)

TNF‐α is a mighty potent proinflammatory cytokine mainly secreted by macrophages that can regulate the inflammatory response and immune cells.^69^ Plasma concentrations of soluble fractions of TNF‐α receptors 1 and 2 (sTNFR1 and sTNFR2, respectively) were found to be increased in diabetic neuropathy patients with T1DM.^50^ In the Cooperative Health Research in the Region of Augsburg F4/FF4 cohort prospective study, it was reported that increased plasma levels of TNF‐α predicted both the onset and progression of DPN.^70^ Compared with nondiabetic mice, TNF‐α^+/+^ diabetic mice have displayed significant impairments in motor and sensory nerve conduction velocities, tail‐flick test, and intraepidermal nerve fiber density, as well as increased expression of NF‐κB p65 and cleaved caspase‐3 in their DRG. Diabetic TNF‐α^−/−^ mice showed no evidence of abnormal nerve function compared to nondiabetic TNF‐α^−/−^ mice.^71^ Increased levels of TNF‐α plasma protein were found in diabetic neuropathic patients and were closely related to neuropathic pain.^72^ Nerve biopsy in patients with painful neuropathy showed high expression levels of TNF‐α.^73^ TNF‐α induces COX‐2 overexpression, which, in turn, generates causes inflammatory changes that lead to DPN.^7^, ^74^ Membrane K^+^‐ion conductivity can also be enhanced in a non‐voltage‐gated manner, resulting in the overexcitation of the whole neuron, resulting in TNF‐α enhancing the tetrodotoxin‐resistant (TTX‐R) Na^+^ current in cultured DRG cells from wild‐type animals compared to those from neuropathic pain model animals.^75^ TNF‐α automatically stimulates its production through G‐protein‐coupled receptors and TNF‐α invertase, induces the expression of miR‐146a, inhibits the expression of TRAK1 and TRAF6, and reduces the expression of Toll‐like receptors,^30^ which results in a series of reactions that lead to the production of IL‐1, IL‐6, NO, and ATP, all of which activate neurons involved in pathological pain.^75^

#### 3.2.2 Interleukin 1 (IL‐1)

IL‐1 is a critical molecule in the immune system, mediating the inflammatory response to various stimuli. IL‐1 produces multiple cytokines and chemokines by activating nuclear factors.^76^ Compared with a control group and type 2 diabetes mellitus (T2DM) group, DPN rats expressed a higher level of IL‐1β.^77^ In activated macrophages, lipopolysaccharide (LPS) activates the pentose phosphate pathway, the serine synthesis pathway, and one‐carbon metabolism, the synergism of which drives epigenetic reprogramming for IL‐1β expression, which operates the proinflammatory phenotype.^78^ IL‐1β plays a vital role in neuropathic pain. Liao YH et al. found that the expression of IL‐1β could be significantly enhanced by activated astrocytes in the spinal cord, which may induce the phosphorylation of N‐methyl‐D‐aspartate receptor (NMDAR) in spinal dorsal horn neurons and improve pain transmission in *db/db* mice.^79^ However, the injection of IL‐1β alone does not induce a nociceptive effect.^80^

#### 3.2.3 Interleukin 6 (IL‐6)

IL‐6 is not only a proinflammatory cytokine but also a pleiotropic cytokine that plays diverse physiological roles, including the regulation of immunity,^81^, ^82^ metabolism,^83^ endothelial function,^84^ and neuronal activity.^85^, ^86^ IL‐6 is a sensitive marker for predicting the progression and severity of type 1 diabetic nephropathy.^87^ Plasma levels of IL‐6 predicted the incidence of neuropathy over 5 years in Chinese diabetes patients.^88^ The KORA F4 study showed increased IL‐6 with MNSI and painful DPN.^89^, ^90^ Another study from Germany revealed that IL‐6 was associated with DSPN and reduced motor NCV.^91^ It is unclear whether IL‐6 is beneficial in developing diabetic neuropathy. IL‐6 mediates pathological pain related to peripheral nerve injury,^92^ and IL‐6 treatment caused marked improvements in several measures of somatic sensory and motor nerve function of conduction velocities, tactile allodynia, and thermal hyperalgesia in experimental diabetes.^93^, ^94^ In contrast, liraglutide treatment reduced the level of IL‐6 in type 1 diabetic patients but did not improve the function of neurons.^95^ IL‐6 may have dual effects under different conditions.^96^

#### 3.2.4 Interferon (IFN)‐γ

IFN‐γ is a lymphokine with diverse but mainly proinflammatory effects on immunocytes and target tissue.^97^ IFN‐γ plays an essential role in the development of neuropathy in nonobese diabetic (NOD) mice, and T‐cell infiltration in the peripheral nervous system of NOD‐B7‐2KO mice is significantly reduced or even blocked due to a lack of IFN‐γ.^98^

### 3.3 Role of chemokines in diabetic neuropathy

Chemokines are a large family of small chemokines that stimulate the migration of cells, especially leukocytes, by signaling through cell surface G‐protein‐coupled heptahelical chemokine receptors.^99^ They are released locally at sites of inflammation and act as signaling molecules in the inflammatory response, which can activate various proinflammatory mediators and induce various inflammatory factors.^80^, ^100^ Regarding the CXC family of chemokines, the expression of CXCL13 in human inflammatory demyelinating nerves is increased.^101^ In B7‐2‐deficient NOD mice that have developed spontaneous autoimmune polyneuropathy, CXCL10 mRNA levels are increased in the sciatic nerve.^102^ According to the results of a meta‐analysis, CCL1, CCL2, CCL4, CCL5, CCL11, CXCL8, CXCL10, and CXCL13 chemokines were significantly higher in patients with T2DM than in controls.^103^ Genetic mapping and gene–phenotype studies have revealed the genetic architecture of T1DM, suggesting that chemokine signaling is also the main pathogen of T1DM.^104^

### 3.4 Role of adhesion molecules in diabetic neuropathy

Diabetes always results in the activation of the endothelium and increases the expression of the adhesion molecules E‐selectin, intercellular adhesion molecule (ICAM‐1), and vascular cell adhesion molecule (VCAM‐1).^105^, ^106^ These adhesion molecules can induce leukocyte recruitment and infiltration into diabetic tissues.^104^ Dekaban and colleagues provided extensive, ample evidence that the upregulation of selectins and cell adhesion molecules on endothelial cells after injury may potentiate destructive neuroinflammation.^107^ Adhesion molecules can be activated by the NF‐κB pathway.^108^, ^109^ Serum VCAM‐1 can reflect inflammatory activity.^110^, ^111^ Studies have shown that connecting adhesion molecule‐A (JAM‐A) plays a nonredundant and novel role in controlling inflammation by regulating the integrity and permeability of the epithelial barrier.^112^ Poorly controlled type 2 diabetes patients have higher sE‐selectin and soluble VCAM‐1 levels.^113^ Diabetic patients with chronic infection have higher levels of VCAM‐1 and tumor necrosis factor receptor 1.^111^

### 3.5 Inflammation‐related signaling pathways

#### 3.5.1 Activation of the NF‐κB pathway

NF‐κB was first identified as a protein that binds to a specific, conserved DNA sequence in the nuclei of activated B lymphocytes, so we named it nuclear factor binding near the κ light‐chain gene in B cells, or NF‐κB.^114^ It is triggered by genotoxic, oxidative, and inflammatory stress and regulates cytokines, growth factors, and genes that regulate apoptosis, cell cycle progression, cell senescence, and inflammation.^115^, ^116^, ^117^

Numerous studies have shown the critical role of the NF‐κB pathway in the pathogenesis of diabetic neuropathy. Activated NF‐κB was observed in the perineurium, epineural vessels, and endoneurium in sural nerve biopsy samples from patients with impaired glucose tolerance and diabetes and model mice.^118^, ^119^ Furthermore, elevated levels of NF‐κB were also observed in Schwann cells cultured in high‐glucose medium compared to those cultured in low‐glucose medium.^120^ NF‐κB is a significant intracellular target of hyperglycemia and oxidative stress.^121^ In hyperglycemia, excessive ROS, iNOS activation, and AGEs can activate the classic NF‐κB pathway.^122^ It was found that the effects of hyperglycemia on ROS formation and NF‐κB activation preceded the stimulation of other systems, which indicated that the activation of NF‐κB was an initial signaling event.^123^ The activation of the NF‐κB signal cascade in experimental diabetic neuropathy increases the expression of NF‐κB, IκB‐α, and phosphorylated IκB‐α and the nuclear translocation of the p65/p50 subunit, promoting the production of proinflammatory cytokines such as IL‐6, TNF‐α, cox‐2, and iNOS, which then initiates the neuroinflammatory response.^124^, ^125^ The neuroinflammatory response causes the proliferation of related inflammatory cells, further enhancing the release of proinflammatory mediators and forming a vicious inflammatory response cycle. Thus, reducing the activity of the NF‐κB pathway may be therapeutically beneficial.

#### 3.5.2 Activation of the MAPK pathway

Mitogen‐activated protein kinases (MAPKs) are a group of intracellular messenger proteins that mainly consist of extracellular signal‐regulated protein kinases (ERKs), p38 kinases, and c‐Jun NH2‐terminal kinases (JNK).^126^, ^127^, ^128^ They may be activated in injured nerves via different mechanisms, which are considered essential media in cell differentiation, apoptosis, and the development of diabetic complications.^127^, ^128^, ^129^, ^130^ MAPKs may be involved in the cellular response to diabetes and activated by hyperglycemia‐induced oxidative stress in the DRGs of rats with streptozotocin (STZ)‐induced diabetes.^131^ Studies have demonstrated that MAPKs significantly contribute to the development of neuropathic pain, and the inhibition of the MAPK pathway can rescue inflammation or relieve pain.^132^, ^133^ The activation of ERKs and p38 is involved in developing the mechanical allodynia observed in the early stages of disease in db/db mice.^134^ Glucagon‐like peptide‐1 receptor (GLP‐1R) agonists were found to prevent nerve dysfunction in the sciatic nerves of diabetic rats via p38 MAPK/NF‐κB signaling pathways independent of glycemic control.^135^ Low‐dose insulin therapy suppresses MAPK signaling and ameliorates peripheral sensory nerve dysfunction in rats with STZ‐induced diabetes.^136^ The AGE/RAGE interaction triggers ERK/MAPK and JNK and activates transcription factors such as CREB and AP‐1, which results in oxidative stress and inflammation.^137^ JNK and p38 pathways promote M1 macrophage polarization, M2 to M1 macrophage transformation, and inflammation. IL‐35 promotes inflammatory progression in DPN rats by inhibiting JNK signal transduction.^138^

### 3.6 The inflammation exacerbates ischemia of nerve cells

Studies have revealed that diabetic peripheral neuropathy is associated with pathological microvascular changes, such as endothelial cell proliferation, basement membrane thickening, and pericyte degeneration. These vascular alterations are closely linked to clinical defects and neuropathology. Researchers have observed reduced neural blood flow and increased resistance of endothelial cells in both human and animal models, leading to an ischemia–hypoxia state of nerve cells. Additionally, compelling evidence suggests hypoxia causes nerve fiber degeneration and damage in diabetic peripheral neuropathy.^139^ In addition, studies have shown that inflammation can worsen ischemia, which is also the case in diabetic peripheral neuropathy. Inflammation causes vascular swelling and constriction, reducing blood flow and increasing hypoxia in nerve cells. This can further harm Schwann cells, essential for nerve cells and myelin sheaths that encase nerve fibers.^140^, ^141^

### 3.7 Proinflammatory changes in bone marrow

Diabetes affects the production of mesenchymal stem cells (MSCs), essential for tissue maintenance and repair. In type 2 diabetes patients, particularly those with osteogenic potential, one study found a reduction in the number of MSCs derived from bone marrow.^142^ Additionally, diabetes can cause proinflammatory changes in the bone marrow that may affect the production and function of various immune cells. One way this occurs is by enhancing the production of proinflammatory cytokines such as TNF‐α, IL‐1β, and IL‐6 in the bone marrow. Proinflammatory cytokines can impact hematopoietic stem cell quiescence, proliferation, and differentiation. For example, TNF‐α induces apoptosis of hematopoietic stem cells and progenitor cells while affecting their self‐renewal/differentiation balance.^143^

Similarly, IL‐1β and IL‐6 regulate survival, proliferation, and differentiation processes within hematopoietic stem cells/progenitors. A study showed that STZ‐induced diabetic mice had significantly increased levels of IL‐1β in their bone marrow, thus suggesting its involvement in regulating hematopoiesis under diabetic conditions. The same study also found a significant correlation between STZ‐induced diabetes and elevated levels of pro‐sexual gene expression within progenitor cells.^144^ Furthermore, oxidative stress caused by diabetes leads to DNA damage/cell death exacerbating inflammatory responses within the bone marrow. These proinflammatory changes may disrupt immune homeostasis by affecting both the bone marrow microenvironment/immune cell production leading to DPN development potentially.

## 4 THERAPEUTIC POTENTIAL OF TARGETING THE INFLAMMATORY RESPONSE IN THE TREATMENT OF DIABETIC NEUROPATHY

Due to the lack of therapies that target underlying nerve damage, there are no effective therapeutic strategies for diabetic neuropathy. Although early and intensive glucose control can reduce the incidence of neuropathy in patients with T1DM,^145^, ^146^ it has little effect in patients with T2DM.^147^ In addition, pain is the most severe consequence of neuropathy. The American Diabetes Association recommends tricyclic antidepressants, anticonvulsants, and opioids, such as tapentadol or oxycodone.^12^ However, only one‐third of patients report at least a 50% reduction in pain after treatment.^148^

As mentioned above, the inflammatory response plays an indispensable role in the pathogenesis of diabetic neuropathy. Therefore, inhibiting inflammation is the new hope for treating or preventing diabetic neuropathy (Table 2).

**TABLE 2:** Anti‐inflammatory agents for diabetic neuropathy.

Drug	Target	Protocol	Effects	Ref.
BAY 11–7082	NF‐κB inhibitor	Rats (1 and 3 mg/kg/day, 2 weeks, p.o.)	Ameliorated abnormal sensory responses and deficits in nerve functions, reduced the expression of NF‐κB, IκB, and p‐IκB, and curbed down the levels of IL‐6, TNF‐α, COX‐2, and iNOS in the sciatic nerve	^149^
JSH‐23	NF‐κB inhibitor	Rats (1 and 3 mg/kg/day, 2 weeks, p.o.)	Reversed the nerve conduction and nerve blood flow deficits in diabetic animals, and reduced the elevated IL‐6, TNF‐α, COX‐2, and iNOS levels	^125^
Melatonin	NF‐κB inhibitor	Rats (3 and 10 mg/kg/day, 2 weeks, p.o.)	Ameliorated the function deficits along with improvement in pain by modulating neuroinflammation via decreasing NF‐κB activation cascade and increasing Nrf2 expression	^150^ ^.^ ^124^
Meloxicam	Selective COX‐2 inhibitor	Rats (1 mg/kg/day, 4 weeks, p.o.)	Prevents diabetes‐induced motor nerve conduction slowing and endoneurial blood flow deficits	^153^
SD‐282	MAPK inhibitor	Rats (15 and 45 mg/kg/day, 1 week, i.p.)	Correction of mechanical allodynia and attenuated flinching behavior during the quiescent period and the second phase of the formalin response in STZ‐diabetic rats	^155^
Naringin	Flavonoids	Rats (40 and 80 mg/kg/day, 4 weeks, p.o.)	Attenuates decreases in nociceptive thresholds; is an endogenous antioxidant and membrane‐bound inorganic phosphate enzyme; dose dependently decreases elevations in oxidative–nitrosative stress, inflammatory mediators, and apoptosis in neural cells.	^158^
Baicalein	Flavonoids	Mice (30 mg/kg/day, 4 weeks, i.p.)	Alleviates nerve conduction deficits and small sensory nerve fiber dysfunction; counteracts diabetes‐associated p38 MAPK phosphorylation, oxidative–nitrosative stress, and 12/15‐lipoxygenase overexpression and activation.	^159^
Genistein	Flavonoids	Rats (3 and 6 mg/kg/day, 4 weeks, s.c.)	Reverts neuropathic pain symptoms and the overexpression of IL‐1β and IL‐6; reverts increases in proinflammation cytokine levels in the sciatic nerve.	^157^

### 4.1 NF‐κB inhibitors

In STZ‐induced diabetic neuropathy model animals, BAY 11‐7082, an IκB phosphorylation inhibitor, ameliorated abnormal sensory responses and deficits in nerve functions, reduced the expression of NF‐κB, IκB, and p‐IκB, and decreased the levels of IL‐6, TNF‐α, COX‐2, and iNOS in the sciatic nerve.^149^ In addition, JSH‐23, an NF‐κB inhibitor, reversed the nerve conduction and nerve blood flow deficits in diabetic animals and reduced the elevated IL‐6, TNF‐α, COX‐2, and iNOS levels.^125^ Furthermore, melatonin ameliorated the functional deficits and improved pain by modulating neuroinflammation by decreasing the NF‐κB activation cascade and increasing Nrf2 expression.^124^, ^150^ α‐Lipoic acid inhibited the activation of NF‐κB and subsequently increased monocyte adhesion to endothelial cells, improving vascular dysfunction and diabetic peripheral neuropathy in rodent models of diabetes.^108^, ^151^, ^152^

### 4.2 COX‐2 inhibitors

Selective inhibition of the proinflammatory enzyme COX‐2 by meloxicam prevented diabetes‐induced motor nerve conduction slowing and endoneurial blood flow deficits in experimental diabetic neuropathy.^74^, ^153^, ^154^

### 4.3 MAPK inhibitors

The p38 MAPK pathway is a signaling pathway within cells crucial in transmitting hyperglycemia‐induced biochemical and metabolic changes. This pathway has been linked to conditions such as diabetic neuropathy. SD‐282, an inhibitor of this pathway, has demonstrated promising results in managing pain associated with diabetic neuropathy.

SD‐282 is a selective inhibitor of p38α MAPK, which belongs to the indole‐5‐carboxamide class. It is 14.3 times more potent against p38α than p38β. Notably, SD‐282 does not affect other closely related kinases, such as jun‐N‐terminal kinase, p38δ, and p38γ, even at concentrations up to 50 mM in human PBMCs. This indicates that the effects of SD‐282 are likely due to its specific inhibition of the p38α MAPK isoform.^155^

In a study examining the effect of SD‐282 on streptozotocin‐induced diabetic rats, it was found that the inhibitor was effective in managing symptoms of diabetic neuropathy. The rats had exhibited symptoms of mechanical allodynia, decreased mechanical thresholds, and C‐ and Aδ‐fiber‐mediated thermal hyperalgesia, all significantly reduced with the administration of SD‐282. This suggests that the p38α MAPK inhibitor can effectively reduce inflammation and pain in diabetic neuropathy, providing a promising therapeutic avenue for this challenging condition.^155^

### 4.4 Flavonoids

Flavonoids are a ubiquitous group of naturally occurring polyphenolic compounds characterized by the flavan nucleus and represent one of the most prevalent classes of compounds in fruits, vegetables, and plant‐derived beverages. Flavonoids are considered health‐promoting and disease‐preventing dietary supplements with antibacterial, antioxidant, and anti‐inflammatory effects. Flavonoids, such as genistein, baicalein, and naringin, were found to alleviate diabetes‐induced neuropathic pain by inhibiting the anti‐inflammatory response.^156^, ^157^, ^158^, ^159^

Although animal experiments have achieved exciting results, most clinical trials have produced disappointing results.^160^ On the one hand, the failures of new drugs may result from multiple mechanisms that contribute to neuronal injury in diabetes. On the other hand, these trials have often been confounded by high improvement rates in the placebo group or other unanticipated effects.

### 4.5 Using a small molecule to regulate Hsp70 and Hsp90 may improve DPN

Diabetes significantly increases inflammatory pathways, promoting the development and progression of DPN. Molecular research highlights the critical role of molecular chaperones, particularly heat shock proteins (HSP), in regulating these pathways and influencing neuro‐pathological defects. Two novel biorexin analogs, KU‐32 and KU‐596, have shown significant therapeutic potential in this context.^141^, ^161^, ^162^

The drug KU‐32 can regulate Hsp90 and Hsp70, making it a promising candidate for treating various defects associated with DPN in animal models of type 1 and type 2 diabetes. These defects include psychological perception, electrophysiology, morphology, and bioenergy. Notably, treatment with KU‐32 improved mitochondrial bioenergy—a crucial factor in nerve health—closely linked to DPN reduction. It is worth noting that this improvement relies on Hsp70 because the drug does not work in mice with type 2 diabetes who lack this molecular chaperone. This underscores the significance of Hsp70 in the pathology and treatment of DPN.^161^

Another novel Biogen analog, KU‐596, has been found to exhibit neuroprotective activity and improve diabetes‐induced hypoalgesia and sensory neuronal bioenergetic defects in a dose‐dependent manner. However, it is essential to note that the drug does not improve these neuropathological defects in type 2 diabetic Hsp70 knockout mice, which further emphasizes the critical role of Hsp70. The anti‐inflammatory effect of KU‐596 appears to be independent of Hsp70, while its impact on reducing regulated reactive oxygen species gene expression depends more on Hsp70. These findings suggest that although targeting molecular chaperones with monologues may be a practical approach to correct neural dysfunction in DPN, normalizing inflammatory pathways through monologues alone may not be sufficient to reverse sensory deficits associated with losing sensation in DPN.^162^

The “heat shock response” (HSR) has become an attractive therapeutic target due to its protective effect against abnormal physiological conditions, stress, and carcinogenesis. Hsp90 is the main regulatory factor of HSR. It represents a promising treatment method that can be used to combat peripheral and central nervous system lesions when regulated by small‐molecule drugs.^163^

Inflammation's interaction with DPN provides a potential pathway for therapeutic intervention. Although existing treatment strategies mainly focus on controlling disease progression and managing symptoms, recent research on molecular chaperones, heat shock proteins, and bioenergetic regulation brings hope for more targeted and effective treatment options. However, further exploration in relevant fields is necessary to translate these findings into clinical applications.

### 4.6 Alleviating Immune Cells to Improve DPN

Glial cells, specifically astrocytes and microglia, work together to promote and maintain neuronal health. They play a crucial role in regulating brain neuronal network activity. Neuroinflammation activates microglia, which promotes the development of pain signal transmission through p38 MAPK activation or P2X4 receptor expression in microglia. Astrocytes are susceptible to hypoxia under acidic conditions caused by diabetes. The spinal cords of diabetic rats consistently report increased activation of microglia.

A study demonstrated that cilostazol administration at both low (10 mg/kg) and high doses (100 mg/kg) could alleviate the dysfunction of spinal cord glial cells induced by streptozotocin. Low doses were sufficient to weaken activated microglia immunoreactivity and restore reduced astrocyte immunoreactivity induced by DM. Cilostazol's potential for regulating essential immune cell function in the central nervous system provides a promising approach for treating inflammation caused by diabetic peripheral neuropathy.^164^

Cilostazol may improve pathological changes related to diabetes in the central nervous system by alleviating functional impairment and activation of microglia and astrocytes. Although these initial findings are encouraging, further research is necessary to clarify cilostazol's mechanism on glial cells and confirm its therapeutic potential for managing diabetic peripheral neuropathy.

## 5 CONCLUSION

The pathogenesis of diabetic neuropathy involves the accumulation or interaction of multiple factors. The presence of “metabolic memory” indicates that obtaining reasonable blood glucose control is insufficient.

Metabolic disorders are more critical than hyperglycemia in the pathogenesis of neuropathy due to T2DM. Despite the evidence that the pathogenesis of DPN may differ between the types of diabetes, only very few studies have investigated potential differences between T1DM and T2DM. In addition, most studies evaluating structural nerve injury are based on T1DM model animals, and only a few studies have used T2DM model animals. Moreover, most studies on neuropathic pain were conducted using nondiabetic animals. This may be why drugs are effective in animal experiments but ineffective in clinical trials. Stronger collaborations among neurologists, endocrinologists, and basic scientists are necessary to solve these problems.

Several lines of evidence link inflammation with the development of diabetic neuropathies. The inhibition of the inflammatory response is effective in treating diabetic neuropathy. Mechanisms of inflammation in diabetic neuropathy include activating the NF‐κB and MAPK/JNK pathways, cytokine and chemokine release, and the recruitment of immune cells. Inflammatory factors have diverse biological effects activating other receptors and pathways that induce nerve injury. Therefore, it is essential to understand the inflammatory cascade changes and select candidate drugs targeting several DPN therapy mechanisms.

## AUTHOR CONTRIBUTIONS

Yifan Cheng, Yinuo Chen, and Kezheng Li contributed equally to this work. Yifan Cheng, Jiali Xie, and Shuwei Liu wrote the manuscript, Yinuo Cheng and Kezheng Li drew the figures and tables, Wenjing LV, Chunyang Pang, and Lingfei Cao reviewed and edited the article, and Binbin Deng is the guarantor of this work and takes responsibility for this review. All authors read and approved the final manuscript.

## FUNDING INFORMATION

This work was supported by the Medical Science and Technology Project of Zhejiang Province (grant number 2022KY506), the National Natural Science Foundation of China (grant number 81901273), and the Science Technology Department of Zhejiang Province (grant number Q21H090076).

## CONFLICT OF INTEREST STATEMENT

The authors declare that they have no competing interests;

## Data Availability

The data that support the findings of this study are openly available in PubMed at https://pubmed.ncbi.nlm.nih.gov.
